# Computer-assisted assessment of the Human Epidermal Growth Factor Receptor 2 immunohistochemical assay in imaged histologic sections using a membrane isolation algorithm and quantitative analysis of positive controls

**DOI:** 10.1186/1471-2342-8-11

**Published:** 2008-06-05

**Authors:** Bonnie H Hall, Monica Ianosi-Irimie, Parisa Javidian, Wenjin Chen, Shridar Ganesan, David J Foran

**Affiliations:** 1 Graduate School for the Biomedical Sciences, UMDNJ, 675 Hoes Lane, Piscataway, New Jersey, USA; 2 Center for Biomedical Imaging and Informatics, 675 Hoes Lane, Piscataway, New Jersey, USA; 3 The Cancer Institute of New Jersey, 195 Little Albany Street, New Brunswick, New Jersey, USA; 4 Robert Wood Johnson Medical School, UMDNJ, 675 Hoes Lane, Piscataway, New Jersey, USA; 5 Department of Pathology and Laboratory Medicine, Robert Wood Johnson Medical School, UMDNJ, 1 Robert Wood Johnson Place, New Brunswick , New Jersey, USA; 6 Department of Medicine and Pharmacology, Robert Wood Johnson Medical School, UMDNJ, 1 Robert Wood Johnson Place, New Brunswick , New Jersey, USA

## Abstract

**Background:**

Breast cancers that overexpress the human epidermal growth factor receptor 2 (HER2) are eligible for effective biologically targeted therapies, such as trastuzumab. However, accurately determining HER2 overexpression, especially in immunohistochemically equivocal cases, remains a challenge. Manual analysis of HER2 expression is dependent on the assessment of membrane staining as well as comparisons with positive controls. In spite of the strides that have been made to standardize the assessment process, intra- and inter-observer discrepancies in scoring is not uncommon. In this manuscript we describe a pathologist assisted, computer-based continuous scoring approach for increasing the precision and reproducibility of assessing imaged breast tissue specimens.

**Methods:**

Computer-assisted analysis on HER2 IHC is compared with manual scoring and fluorescence in situ hybridization results on a test set of 99 digitally imaged breast cancer cases enriched with equivocally scored (2+) cases. Image features are generated based on the staining profile of the positive control tissue and pixels delineated by a newly developed Membrane Isolation Algorithm. Evaluation of results was performed using Receiver Operator Characteristic (ROC) analysis.

**Results:**

A computer-aided diagnostic approach has been developed using a membrane isolation algorithm and quantitative use of positive immunostaining controls. By incorporating internal positive controls into feature analysis a greater Area Under the Curve (AUC) in ROC analysis was achieved than feature analysis without positive controls. Evaluation of HER2 immunostaining that utilized membrane pixels, controls, and percent area stained showed significantly greater AUC than manual scoring, and significantly less false positive rate when used to evaluate immunohistochemically equivocal cases.

**Conclusion:**

It has been shown that by incorporating both a membrane isolation algorithm and analysis of known positive controls a computer-assisted diagnostic algorithm was developed that can reproducibly score HER2 status in IHC stained clinical breast cancer specimens. For equivocal scoring cases, this approach performed better than standard manual evaluation as assessed by ROC analysis in our test samples. Finally, there exists potential for utilizing image-analysis techniques for improving HER2 scoring at the immunohistochemically equivocal range.

## Background

### Clinical Introduction

The human epidermal growth factor receptor 2 (HER2) gene on chromosome 17 is amplified in 20% to 30% of breast cancer patients [1]. The protein product is a transmembrane receptor tyrosine kinase whose overexpression in breast cancers is predominantly due to HER2 gene amplification [2]. The proper evaluation of HER2 status is crucial to patient care as its overexpression and/or amplification is associated with less favorable clinical outcomes and relatively poor response to certain treatments [3,4]. Moreover, with the development of effective targeted therapies such as Trastuzumab (Herceptin), a monoclonal antibody to HER2, which are effective only in tumors with HER2 overexpression, the clinical ramifications of accurately assessing HER2 status is further underscored. Thus, accurate HER2 assessment is vital to the identification of breast cancer patients who would benefit from anti-HER2 therapy.

Given the clinical impact of HER2 overexpression, there has recently been a heightened emphasis on reliably assessing HER2 status in breast cancers [5-9]. Guidelines have been issued jointly by the American Society of Clinical Oncology (ASCO) and the College of American Pathologists (CAP) in order to establish protocols for evaluating HER2 status that involve both immunohistochemistry (IHC) and fluorescence in situ hybridization (FISH) [6,10]. Recently a new standard for assessing HER2 expression was instituted that requires that both IHC and FISH be conducted when scores from either fall into the newly established equivocal ranges. ASCO/CAP recommended that an IHC score of 2+ or a FISH score (ratio of HER2/CEP17 genes) of 1.8–2.2 be considered equivocal and that the specimen be graded with the complementary modality, or repeated in the case of FISH. (An alternate option is to reassess the immunohistochemistry at a reference laboratory.)

In light of the pressing need for accurate determination of HER2 overexpression, the authors of the above ASCO/CAP study have acknowledged the use of image analysis as a viable approach for assessment. Previous work with image analysis systems have focused on the validation of commercially available technology in the clinic by demonstrating greater accuracy in quantification of HER2 immunohistochemistry when compared to manual interpretation [4,11-13], and reduction in inter-observer variability with systems such as ACIS (automated cellular imaging system) [13]. However, due to the proprietary nature of such systems, the technical processes that lead up to a HER2 score are often not transparent to the pathologist. This may lead to interpretation difficulties as the exact tissue features being quantified and the methods used to perform the HER2 assessment have not been made available for peer review. For instance, the pathologist has little information regarding how well and to what extent the image analysis software is able to quantify membrane staining. Furthermore, the pathologist does not have access to the manufacturer's criteria or data that established the universal "cutoff score" for HER2 positivity [11]. This can be problematic as cutoff values are not necessarily applicable across laboratories due to variations in staining characteristics which can arise as a result of different choices in antibodies, laboratory equipment, and fixation procedures [14]. Veiling the relevant technical information that is used to render clinical decisions is somewhat inconsistent with the current environment of evidence-based medicine. In the face of these pressing concerns, to our knowledge, only one commercial system has revealed some basic outlines of the image processing methods behind their commercially based system [15].

Furthermore, the cost of performing the analysis may actually serve as a barrier to mainstream adoption of this technology in practice. Skaland et. al. has contributed valiantly in this realm by using readily available (and free) NIH image processing software to analyze images of HER2 stained breast cancer specimens [16]. However, these algorithms use generic image processing methods not designed specifically for these images. For instance, no morphologically based operators are used to detect stained membrane pixels, but rather a simple intensity threshold is used. Therefore, only darkly stained images could be analyzed in their experiments. As a result, we have tried to introduce algorithms specifically tailored to this application.

In this manuscript, we describe the design, development and evaluation of a new suite of algorithms to assist in increasing the precision (i.e. number of scoring levels) at which HER2 immunostains are quantified. We provide a detailed description of the workflow and associated processes that are used to evaluate HER2 stained breast slides and compute the HER2 expression score. We describe three clinically based image features used in our system and compare their performance with manual scoring techniques in assessing specimens. The performance results of the computer-assisted quantification algorithms based on the newly developed image features are then compared with manual scoring using Receiver Operator Characteristic (ROC) analysis. In addition, the adequacy of the quantification algorithm is crucially examined in the equivocal scoring ranges in order to demonstrate its applicability in the laboratory. Finally, we discuss the capacity of the system to be optimally calibrated for the specific conditions of the given laboratory. By increasing the precision with which HER2 immunohistochemistry is quantified, we anticipate that breast cancer patients who might benefit from anti-HER2 therapy will be accurately identified, while the remaining subpopulation of patients will be spared a costly and potentially harmful treatment.

### Designing the Computer-Assisted Quantification Scheme

In order to design a clinically relevant computer-based quantification scheme, we first begin by examining the current methods that are used routinely in the laboratory. Briefly, the pathologist focuses on two primary features to arrive at a HER2 score: the quality of membrane staining (since HER2 is a receptor functioning at the cytoplasmic membrane) and the percent of invasive tumour cells stained (Table 1). In this first attempt at developing a new approach for conducting computer-assisted quantification of specimens, we will address and evaluate the performance of this system based on steps A, B, C, E, and F as shown in Table 1. In this computer-assisted framework, the pathologist will perform Steps A and B. A membrane isolation algorithm will be introduced to account for Step C, and an approximation method will be used to account for Step E. In addition, guided by the fact that the staining characteristics of the control are taken into account during the manual assessment of immunostains (Step F) [17], we introduce computer-assisted analysis of a known positive control (V) into our computations. This is a feature that is not utilized in most commercial systems. Step D is an area left for future investigation.

**Table 1:** Translating Manual HER2 Assessment into Computer-Related Tasks

Pathologist	Computer
A. Visually examines tissue	I. Captures digital image of relevant area of tissue
B. Locates invasive carcinoma	
C. Examines intensity of stain at cytoplasmic membrane	II. Identifies stained membrane areas (pixels) and quantifies intensity on 0–255 scale
D. Examines completeness of membrane staining	III. Quantifies percentage of membrane staining per cell
E. Examines percent positive cells	IV. Counts positive cancer cells and divides by total number of cancer cells
F. Compares with positive tissue controls	V: Takes digital image of control, quantifies control, and uses it to normalize patient HER2 score.

## Methods

### Overview of Computer-Assisted Quantification Scheme

**Figure 1:**
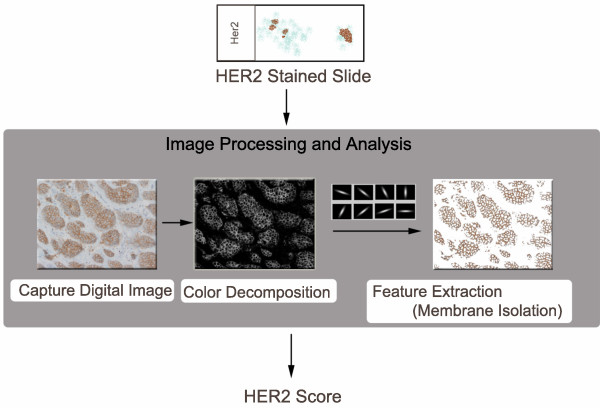
**Overview of computer-assisted image analysis scheme**. The process of transforming a slide into a score begins with the pathologist capturing a digital image. A color decomposition algorithm is performed in order to isolate the DAB stain (brown areas) from the hematoxylin stain (blue areas). Next, a membrane isolation algorithm, utilizing a set of bar filters, isolates the relevant stained membrane pixels. Finally, based on these pixels, a HER2 score can be computed. The process is repeated in order to take into account the positive control.

The workflow of the quantification scheme is outlined in Figure 1. Standard formalin-fixed, paraffin-embedded HER2 IHC stained diagnostic sections of breast tissue are visualized using diaminobenzidine (DAB) and counterstained with hematoxylin. A relevant microscopic field of the tissue is digitally imaged by a certified pathologist. All pixels which are stained with DAB, indicating areas of HER2 protein, are isolated by utilizing a color decomposition algorithm that was previously described [18]. Subsequently, image-based quantification of HER2 staining proceeds through identifying stained membrane regions using a filter-based algorithm (II) and comparison with positive controls (V). Lastly, we provide a gross estimate of the percentage of positive tumor cells (IV) by factoring the percentage of stained area (expressed in pixels) into the feature analysis.

### Case selection, processing, and manual grading

The specimens used in these studies consisted of 99 breast cancer cases that had been diagnosed between January 2005 and March 2007 and stored in archives at the Department of Pathology and Laboratory Medicine at Robert Wood Johnson University Hospital, New Brunswick, and N.J. During this period of time it was standard procedure for both assays to be performed. Cases which had received an IHC score of 0 or 1+ were limited in order to enrich the amount of 2+ cases (considered equivocal) to approximately 25% of the data. The purpose of this experimental design was that since 2+ cases are considered equivocal, they represent the patient population that would tend to benefit most from the use of computer aided quantification. Specimens with significant mechanical crushing and sectioning artifacts were omitted from the study to arrive at the final 99 cases. Both invasive ductal and lobular carcinomas were included in the experiments.

Immunohistochemical staining was performed at the Department of Pathology and Laboratory Medicine at Robert Wood Johnson University Hospital utilizing an automated immunostainer, Ventana BenchMark IHC/ISH system (Ventana Medical Systems, Inc. Tucson, AZ). Ventana PATHWAY HER-2 (clone CB-11) was used for the primary detection of c-erbB-2 antigen in sections of formalin fixed, paraffin embedded tissue. A known 3+, FISH positive control was fixed on each slide along with the patient sample in order to analyze both under identical staining conditions.

Manual immunohistochemical grading was performed by a board certified surgical pathologist (P.J.) at Robert Wood Johnson University Hospital according tithe Scoring Guide for the Interpretation of Ventana Pathway HER2 Staining of Breast Carcinomas [19]. Briefly, if no membrane staining was observed the specimen was scored as 0. Faint, partial staining of the membrane was scored as 1+. Weak complete staining of the membrane, >10% of cancer cells was scored as 2+. Intense complete staining of the membrane, >10% of cancer cells, was scored as 3+. A score of 2+ or greater is considered positive according to the manufacturer's instructions.

Specimens were sent to Genzyme Genetics (Westborough, MA) for FISH analysis and the ratio of discrete signals for the HER2 gene and centromere probe for chromosome 17 (CEP17) was reported. A ratio of HER2 gene/CEP17 ≥ 2.3 was considered positive for HER2 overexpression.

### Image Selection and Capturing

A board certified pathologist (P.J.) delineated the region of invasive carcinoma, and then a resident pathologist (M.I.) used a robotic microscope to digitally acquire images at 20× magnification within the specified boundaries. The control from each slide was also digitized at 20× magnification. All images are taken with an Olympus AX70 microscope (Olympus America Inc., Melville, NY) equipped with a Prior six-way robotic stage and motorized turret (Prior Scientific, Inc., Rockland, MA) and Olympus DC330 720-line 3-Chip video camera at 1360 × 1024 pixel resolution and a fixed exposure time of 1/600 s. This allows the digital images to be captured through red, green, and blue channels, and thus, span the entire visible range.

### Color Decomposition

**Figure 2:**
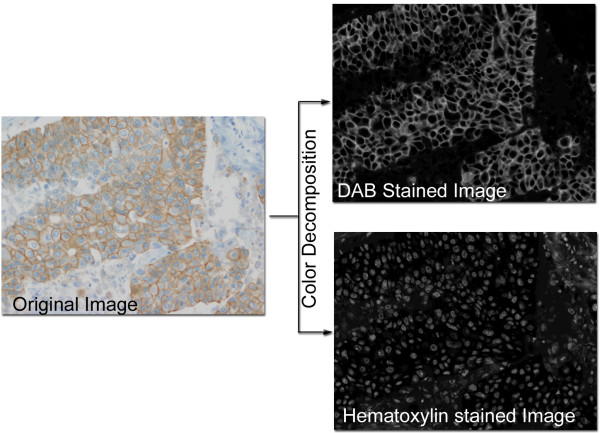
**Color Decomposition**. This is an example image of a breast cancer specimen stained for HER2 using DAB as the chromagen and counterstained with hematoxylin. Color Decomposition [18] is applied to the original image and as a result, a gray scale DAB stained image plane and a hematoxylin stained image plane is produced.

The DAB (HER2) stained regions were isolated from the hematoxylin counterstain in the digital image of the patient sample and control images through the use of color decomposition (Fig. 2) [18]. Based on a polar transformation in a color space where the hematoxylin and DAB colors are laid out on a super-plane, color decomposition enables the digital image to be separated into the hematoxylin stained image and the DAB stained image of the tissue. In a sense, this algorithm attempts to describe light absorbance from certain color ranges (DAB and Hematoxlyin) without the use of expensive spectral imaging equipments.

### Membrane Isolation Algorithm (MIA)

**Figure 3:**
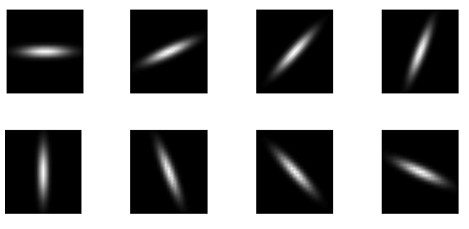
**Rotationally invariant bar filter**. Eight bar filters, each successively rotated 2π/8 degrees, are used to detect the cytoplasmic membrane through a convolution with the DAB stained image. The Gaussian-based bar filter is centered on a 25 × 25 pixel base, with (σ_x_, σ_y_) = (5,1), thus creating an elongated bar. The filters as a set are rotationally invariant as only the maximum response from the convolution is chosen. A view of these filters as an image where higher values are depicted brighter is shown.

In order to isolate the membrane stained regions, Otsu's method [20] was used to preprocess the DAB image by automatically removing background noise. Subsequently, a rotationally invariant bar filter was used to detect membranes throughout the DAB stained image. The rotationally invariant filter was created using a set of eight Gaussian based bar filters rotated 2π/8 degrees apart (details in Fig. 3). Similar to [21], rotational invariance is achieved by keeping only the maximum response from the convolution of these 8 filters with the DAB image. Various thresholds were then applied to the maximum response from convolution with the bar filters. The thresholds (k = [1,5,7,9,12,15,20]) used to isolate membrane pixels were evaluated based on their ROC performance. In the results below, only k = 15 data is shown. ROC data were calculated for all thresholds k (see Additional file 1). Membrane isolation results on lighter stained specimens are upon inspection visually more satisfying (see Additional File 2). However experiments conducted using lower thresholds for lighter stained images and higher thresholds for darker images, provided no added benefit in terms of increasing AUC (see Additional file 3).

### Image Features and HER2 Score

In these experiments, three scoring features based on mean intensity were reported. The first image feature used to calculate a score is *M*_*p *_the mean intensity of the patient's stained membrane regions,

(1) $$M_{p}={\bar{I}}_{p}$$

where *I *is the set of intensities derived from the pixels retained from performing the MIA on *p *the patient tissue; the bar denotes the mean function.

2. The second image feature is *M*_*n *_which is *M*_*p*_normalized by the positive control

(2) $$M_{n}=\frac{M_{p}}{M_{c}}.$$

*M*_*c *_is defined similarly to *M*_*p *_except that the calculation is based on *c *the control tissue $M_{c}={\bar{I}}_{c}$.

3. The third feature *M*_*a *_adds a coefficient *d/N*, to *M*_*n*_

(3) $$M_{a}=\frac{d}{N}M_{n}$$

where *N *is the total amount of pixels in the image, *d *is the number of DAB stained pixels after pre-processing. The coefficient *d/N *is used as an approximation for the percentage of stained cells. Results were also gathered for median-based features; however, results were similar, and therefore not included in this manuscript.

### Hardware and Implementation

An Intel Core 2 Duo T7400 (2.16 GHz) computer with 1.0 GB memory was used for processing each image. The color decomposition algorithm was implemented in Java. All other processing was implemented utilizing Matlab (The MathWorks, Inc. Natwick, MA) code. The color decomposition and membrane isolation together took approximately 10–20 seconds per image to complete.

### Statistical Analysis

Receiver Operator Characteristic curve analysis was used to compare the accuracy of the manual and automated computerized methods for grading IHC. FISH assays were used as an independent means for assessing IHC scores. The area -under- the -curve (AUC) was calculated for each ROC curve utilizing the trapezoidal rule [22] in order to compare the accuracy of each method as a test for FISH positivity. Since the data are derived from the same patient cases, p-values are calculated using a nonparametric method based on the Mann-Whitney-U Statistic [22]. The McNemar Test for correlated proportions [23,24]was used to calculate the p-values for differences between sensitivity or specificity.

### Institutional Review Board Approval

Institutional Review Board approval was obtained for this retrospective study through protocol (IRB #5381).

## Results

### Case Selection

Table 2 summarizes the HER2 expression as determined by FISH and manual grading for these 99 cases. Similar to [25], our data included cases in which the gene was found to be amplified, but protein levels determined by IHC were not overexpressed (IHC < 2+), and cases where IHC = 3+, in which there was no gene amplification.

**Table 2:** Summary of HER2 manual grading and FISH scores

*FISH Score*	*IHC = 0*	*IHC = 1*	*IHC = 2*	*IHC = 3*	*Total*
≤ 1.7	25	22	15	6	68
1.8 – 2.2	1	1	2	0	4
≥ 2.3	2	3	5	17	27
Total	28	26	22	23	99

### Analysis of Specimens using Stained Membrane Regions with and without controls

**Figure 4:**
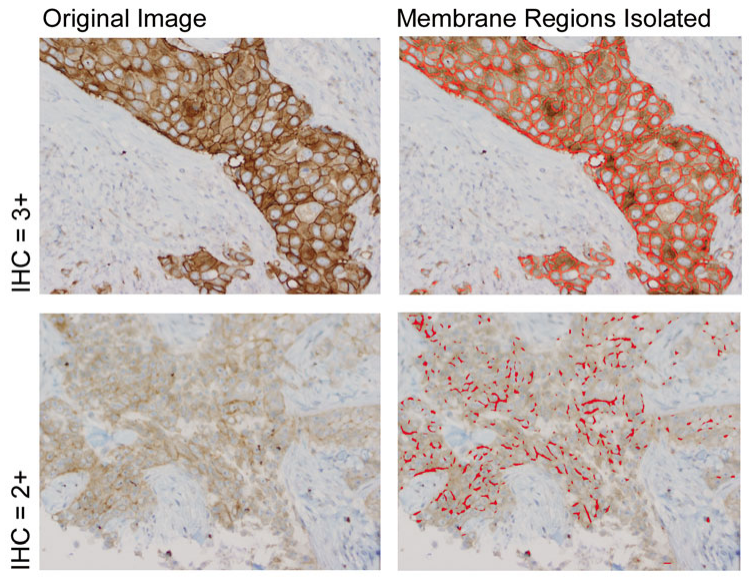
**Results of the membrane isolation algorithm**. Two examples of HER2 images (left column) taken from a manually graded 3+ case (top row) and 2+ case (bottom row) with their isolated membrane regions (right column). Images are captured at 20× magnification and stained with DAB and hematoxylin.

Two examples of HER2 stained images (Fig. 4) are shown. The pixels chosen after applying the membrane isolation algorithm are visualized by red areas overlaying the original image. It is important to note that biologically irrelevant cytoplasmic pixels were not selected since the HER2 receptor is a transmembrane protein functioning at the cytoplasmic membrane.

**Figure 5:**
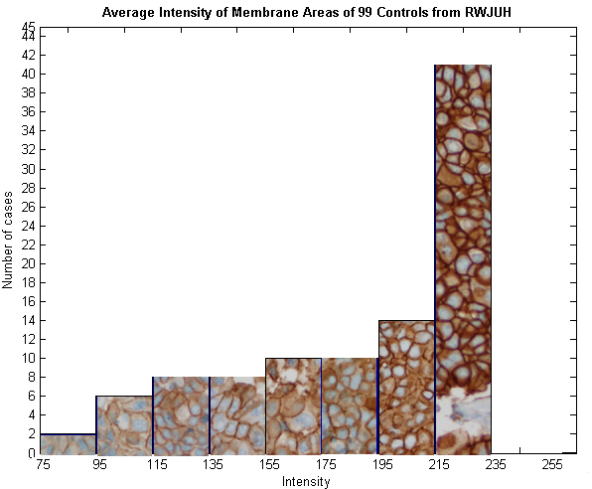
**Variations on the Membrane Staining Intensities of Positive Control Specimens**. This is a histogram showing the amount of specimens in each range of intensities. Each bar contains cases that lie within a 20 unit range of intensities. Though most positive controls stain darkly (215–235), there is a significant number of cases in which staining intensity deviates markedly from the mode. Consequently, features that take into account the intensity of the positive control are used in this study.

Figure 5 shows the variations in staining intensities of the membrane regions of known HER2 positive controls (taken from breast specimens that are FISH positive and previously manually scored as 3+) used in this study. Though most control specimens stain dark, there is indeed variation in intensity which should be taken into account when computing a HER2 score. Hence, we have chosen to derive the *M*_*n *_feature which specifically takes into account the intensity of the positive control.

**Figure 6:**
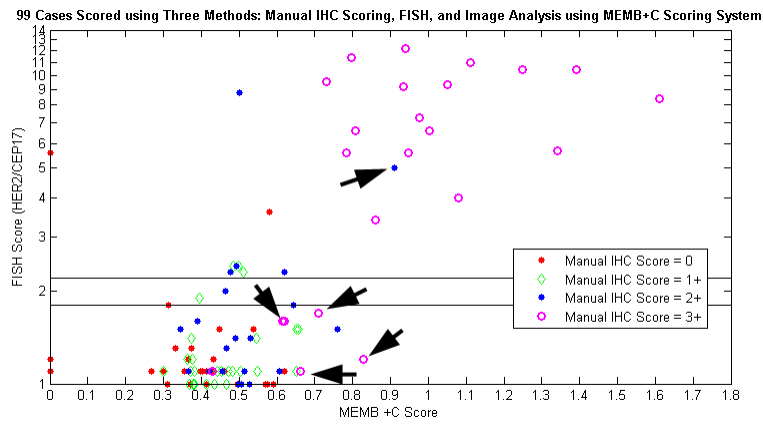
**Simultaneous view of three scoring methods: *M*_*n*_, FISH, and Manual**. Plot of mean intensity of stained membrane regions normalized by control (*M*_*n*_) versus genetic amplification (FISH). Different colors denote manual scores. Arrows depict example cases whose classification was enhanced due to image-based analysis.

Figure 6 displays the results of three scoring methods: user-guided image analysis based on *M*_*n*_, FISH, and standard manual scoring. It is shown that the image-based system is capable of delineating two groups of patients: those with high *M*_*n *_scores and gene amplification, and those with low *M*_*n *_scores and no amplification. The classification of several cases has improved with computer-assisted image analysis (arrows). When image analysis was performed, four IHC 3+ cases (but FISH-) are clustered with the non-amplified group, while a 2+ case with genetic amplification is grouped with other FISH positive cases. Also, it is important to note that, the *M*_*n *_score for some cases was computed to be beyond a ratio of one, indicating that these patient tissues stained darker than the control tissue, which possibly suggests a greater concentration of HER2 protein than the control tissue.

**Figure 7:**
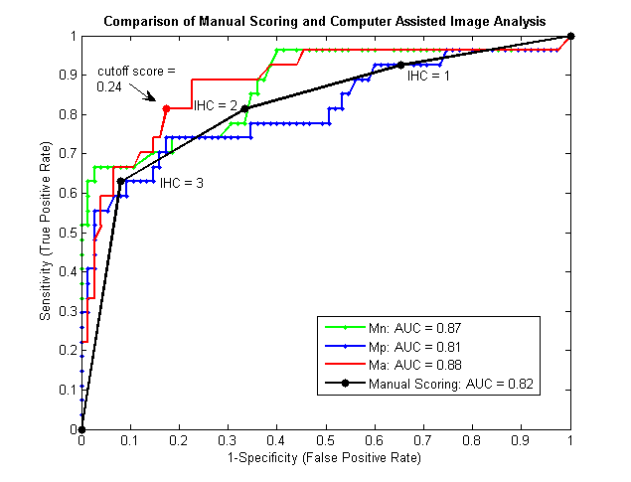
**ROC Curves comparing *M*_*n*_, Manual Scoring methods with Computer Assisted Image Analysis**. These ROC curves report the sensitivity and specificity across all potential cutoff points for positive genetic amplification (≥ 2.3). In this plot, the quantitative use of controls (*M*_*n*_)is shown to significantly increase the area under the ROC curve when compared to tissue analysis alone (*M*_*p*_). The arrow denotes a point on the *M*_*a *_curve where the false positive rate is significantly reduced compared to the 2+ point on the Manual Scoring curve. The AUC's of computer-assisted methods *M*_*n *_and *M*_*p *_are statistically similar to that of manual grading; only *M*_*a *_is statistically different.

In order to quantitatively compare these scoring systems, a ROC curve was generated comparing the diagnostic reliability of the *M*_*n *_and *M*_*p *_features with that of manual scoring (Fig. 7). The optimal ROC curve is one that approaches the upper left-hand corner, with an AUC of 1. Our results show that the area under the *M*_*n *_curve (0.87) is significantly greater than the *M*_*p *_curve (one tailed, p < .05), indicating that quantitatively utilizing the control provides a better diagnostic test than analyzing only patient tissue. When the AUC of the *M*_*n *_curve was compared to that of manual assessment, no significant difference was found (two tailed, p > .14).

### Analysis of Specimens using Stained Membrane Regions incorporating Both Controls and Percent Stained Area

The results from Figure 7 indicated that the performance of the system based on *M*_*n *_score was similar to that of manual scoring. Consequently, the percent stained area was incorporated into the *M*_*a *_feature through an addition of the *d/N *coefficient. The *M*_*a *_ROC curve (red line) is compared to the ROC curve generated from manual scoring (black line, Fig. 7). As evident in this plot, the AUC from the *M*_*a *_feature is significantly greater than that derived from manual scoring (p < .05). More importantly, the difference between the two scoring systems is most pronounced at the clinically crucial point: the equivalent score of 2+. The arrow (Fig. 7) denotes an example *M*_*a *_cutoff point that has a statistically lower false positive rate than that derived by using the 2+ cutoff score.

### Quantification of Equivocal Cases

**Figure 8:**
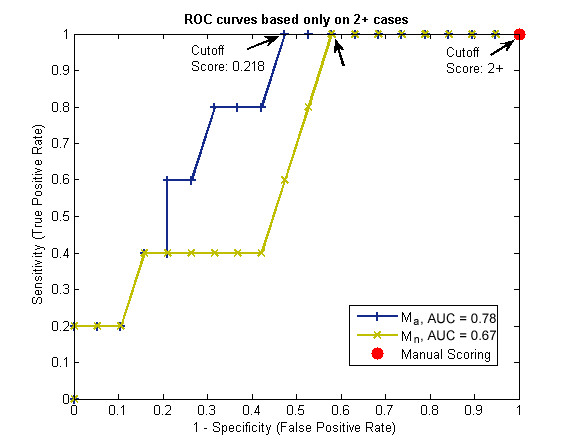
**ROC curves based solely on equivocal 2+ cases for three scoring methods: *M*_*a*_, *M*_*n*_, and Manual Scoring**. The two left most arrows indicate significant decreases in false positive rate when using computer-assisted image analysis techniques. This may potentially show promise in reducing those patients who would otherwise be needlessly exposed to treatment toxicity. The reduction may be attributed to not only the features used, but also the greater ability of computer-assisted analysis to differentiate more than just one cutoff point in the 2+ equivocal range.

Lastly, these computer-assisted scoring methods can be engineered to perform optimally on equivocal cases. Fig. 8 reports the ROC curve for the scores generated by Ma and *M*_*n *_based solely on the 22 manually scored 2+ cases. A specific true positive or false positive rate based on clinical needs can be selected by plotting this ROC curve. For 100% detection of FISH amplified cases (sensitivity of 1), note how the computer-assisted scoring system can significantly reduce false positive rates (arrows) for IHC = 2+ cases (p < .05), as Ventana scoring guidelines considers all 2+ cases weakly positive.

To estimate a robust classification performance of the system on only these 2+ cases, an iterative leave-one-out classification experiment was performed. The cutoff point to determine FISH positivity was based on twenty-one training samples. This cutoff point was chosen as the maximum cutoff point yielding 100% sensitivity (i.e. fix sensitivity at 100% while maximizing specificity). Then the novel case not used in training was scored based on the cut-off score. Correct was defined as either above the cut-off and FISH ≥2.3 (a true positive decision), or below the cut-off score and FISH <2.3 (a true negative). Repeating the experiment 1000× with a randomly chosen test case, we arrived at an estimate of the system's classification performance on 2+ cases. From this experiment, the 2+ test case was correctly classified **64% **of the time, whereas manual scoring was only able to correctly classify **23% **of the cases (5/22: 5 of the 22 2+ cases, which are considered positive, were also FISH positive). There was a reduction false positive rate (percentage of FISH negative cases considered FISH positive) from **100% **to **31%**, as 100% of manually scored 2+ cases are considered by Ventana scoring guidelines to be positive. Lastly, the false negative rate was only **4%**.

## Discussion

In this retrospective study of 99 cases of breast carcinoma, we have demonstrated the processes and methods behind the design, development, and evaluation of an image-based, computer-assisted HER2 immunohistochemistry quantification scheme. The result was an introduction of a membrane isolation algorithm and the quantitative use of positive staining controls. The membrane isolation algorithm in combination with the quantitative use of controls (*M*_*n *_feature) was shown to be superior to analyzing the patient tissue alone (*M*_*p *_feature). This improvement is hypothesized to stem from the unique staining conditions of each slide that is reflected in both the patient and control sample. Furthermore, this clinically based combination (*M*_*n*_) demonstrated an ability to correlate with gene amplification results that was similar to that of manual assessment.

With the establishment of new guidelines defining the equivocal IHC scoring range of 2+, it was hypothesized that computer-assisted image analysis may be useful in assisting in the manual interpretation of these cases. As a result, we explored the addition of an area coefficient to the *M*_*n *_feature to form the *M*_*a *_feature. Using this feature, significant reduction in false positive rate was evidenced especially in the 2+ intensity region (Fig. 7), along with an overall greater AUC than manual grading. A greater AUC is interpreted as a greater probability that a randomly selected FISH positive case would have a higher score than a randomly chosen FISH negative case. In addition, because the computer can distinguish varying levels of intensity and area *within *this 2+ category, we chose a specific point on the ROC curve derived solely from 2+ cases that yielded 100% sensitivity of FISH positive cases, while minimizing the amount of false positive cases (Fig. 8). This produced a cutoff point at a clinically desirable sensitivity and specificity that was selected according to our institutional staining variations. Leave-one-out cross validation experiments based on this cutoff showed promising improvement for equivocal cases when compared to manual scoring. As all 2+ cases are considered positive by Ventana scoring methods, usage of the *M*_*a *_feature to analyze HER2 immunohistochemistry shows the possibility of false positive rate reduction, which would lead to the reduction of patients exposed to unnecessary treatment toxicity. One advantage of our approach to measuring HER2 staining is that unlike clinical scoring, which has the ability of differentiating only 4 scoring levels, computer-assisted quantification outputs a nearly continuous variable, for which fine tuned cut-offs can be identified.

Previously published systems differ in many respects from our proposed algorithms. Firstly, many commercially available systems have a universal cutoff score for HER2 overexpression that is not optimized for a given laboratory, in spite of well established variations in intra-laboratory conditions [14]. However, statistical analyses based on the data collected from that particular institution can provide a meaningful and institutional-specific, cutoff value for immunostains such as HER2 [17]. ROC analysis illustrates the benefits of adjusting for such variations leading to improved sensitivity and specificity. With this information, the selection of a particular cutoff score can be chosen according to each institution's operating parameters, enabling pathologists to estimate the likelihood that a certain score is correct based on the sensitivity and specificity of the chosen cutoff point. In addition, both commercial and noncommercial systems [16] have thus far neglected to include the quantitative analysis of positive HER2 controls in their calculation of a HER2 score. As evidenced in our study, there is sometimes marked variation in staining intensity which is often accounted for in manual scoring techniques, but neglected in published computer-assisted methods. Consequently, our algorithms specifically take into account this variation in staining. And lastly, our algorithms are morphologically based (unlike [16]), demonstrate improvement in scoring 2+ cases, and freely available (unlike many proprietary commercial systems) to further research and improvement.

In the future, research efforts may be concentrated in defining image features that better reflect percent of cancer cells stained (Table 1, Step E) or completeness of membrane staining (Table 1, step D), and providing segmentation algorithms that delineate the invasive component of the carcinoma (Table 1, step B). Indeed, the development of the image-based algorithms required to fully assess HER2 immunohistochemistry is only in its nascent stages, and what we have demonstrated here is only a first step towards that goal. It is hoped that with the combination of features that accurately quantify HER2 expression and the ability to distinguish not just 4 classes of HER2 immunostaining, but rather a continuum, the correlation between HER2 overexpression and response to trastuzumab therapy can be enhanced.

## Conclusion

This work has demonstrated the promise of developing clinically based algorithms to assist in the quantification of the HER2 immunostain. A membrane isolation algorithm was developed that can be readily applied to quantification of membrane stained images. In addition, quantitative use of positive staining controls was shown to significantly increase the diagnostic ability of the features used to quantify HER2 membrane staining. The *M*_*n *_feature used showed similar ability to predict FISH positivity when compared to manual scoring. Furthermore, potential improvement in specificity for the 2+ specimens was demonstrated using the Ma feature. Lastly, by using ROC analysis, we indicated a process by which an institution can derive a cutoff score for computer-assisted image analysis systems that can account for their unique laboratory staining conditions. In the future, we hope to validate this method by analyzing a larger series of independent samples for which both IHC and FISH scores of HER2 are available.

Indeed, we have shown that computer-assisted image-analysis can enhance the degrees to which quantification of immunohistochemistry can be achieved, and improve the correlation of IHC with genetic amplification. Ultimately, much investigation remains to be done in accurately determining which patients not only overexpress HER2, but also, which, in the end, will respond to therapy.

## Competing interests

The authors declare that they have no competing interests.

## Authors' contributions

BHH designed the study, wrote the membrane isolation algorithm, carried out the image analysis, performed the statistical analysis, and drafted the paper. MI–I contributed intellectually by suggesting the quantitative usage of controls, and assisted in gathering images and patient data. PJ contributed intellectually by focusing the study on 2+ cases, reviewed all images, and assisted in gathering patient data. WC provided the color decomposition algorithm and critically reviewed the manuscript and experiments. SG conceived of the study and intellectually contributed to the discussion section. DJF provided funding, critical review of paper, and participated in the coordination of the study. All authors read and reviewed the final draft of the manuscript.

## Pre-publication history

The pre-publication history for this paper can be accessed here:

http://www.biomedcentral.com/1471-2342/8/11/prepub

## Supplementary Material

Additional file 1 **Selecting the optimal threshold for membrane pixels**. Although lower thresholds can produce visually more satisfying membrane isolation results for weaker staining cases (especially IHC = 1+), responses greater than 15 produced the greatest area under the ROC curve using the *M*_*n *_feature, and thus the k = 15 threshold was selected to produce the results in these experiments. Click here for file

Additional file 2 **Membrane Isolation Algorithm results using both high and low thresholds**. These are the results from the membrane isolation algorithm using 2 different thresholds k = 15, and k = 5. These are specimens which stained less intensely. Click here for file

Additional file 3 **ROC curves using Membrane Isolation Algorithm based on k = 15 and a combination (k = 15, k = 5)**. This is a comparison of ROC curves based on different Membrane Isolation Algorithm (MIA) variations. Since lighter cases showed enhanced membrane isolation detection when lower thresholds were used, a MIA using k = 5 threshold for lighter images and k = 15 for darker images was evaluated (green line). However, this did not improve AUC in comparison to one universal threshold (black line), and consequently, only one threshold (k = 15) was used in the results of this manuscript. It is interesting to note that the combination threshold used had very similar results to manual scoring (blue line). Click here for file
