# Supplementary figures and images for: Computer-assisted assessment of the Human Epidermal Growth Factor Receptor 2 immunohistochemical assay in imaged histologic sections using a membrane isolation algorithm and quantitative analysis of positive controls
Source: BMC Med Imaging. 2008 Jun 5;8:11. doi: 10.1186/1471-2342-8-11 (PMC2447833; doi:10.1186/1471-2342-8-11)

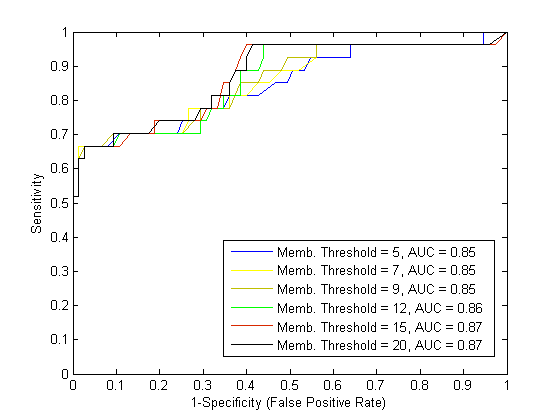

Supplement: Additional file 1 — Selecting the optimal threshold for membrane pixels. Although lower thresholds can produce visually more satisfying membrane isolation results for weaker staining cases (especially IHC = 1+), responses greater than 15 produced the greatest area under the ROC curve using the Mn feature, and thus the k = 15 threshold was selected to produce the results in these experiments. [file 1471-2342-8-11-S1.tiff]

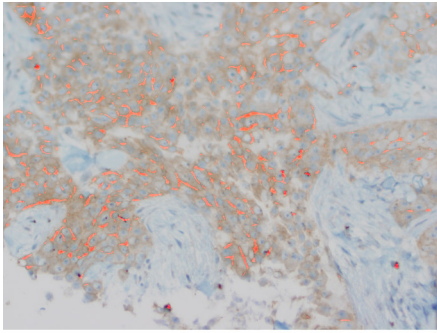

Threshold  $k = 15$

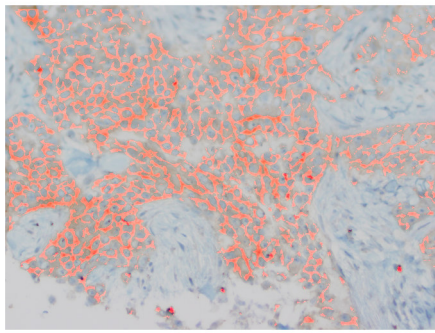

Threshold  $k = 5$

Supplement: Additional file 2 — Membrane Isolation Algorithm results using both high and low thresholds. These are the results from the membrane isolation algorithm using 2 different thresholds k = 15, and k = 5. These are specimens which stained less intensely. [file 1471-2342-8-11-S2.pdf]

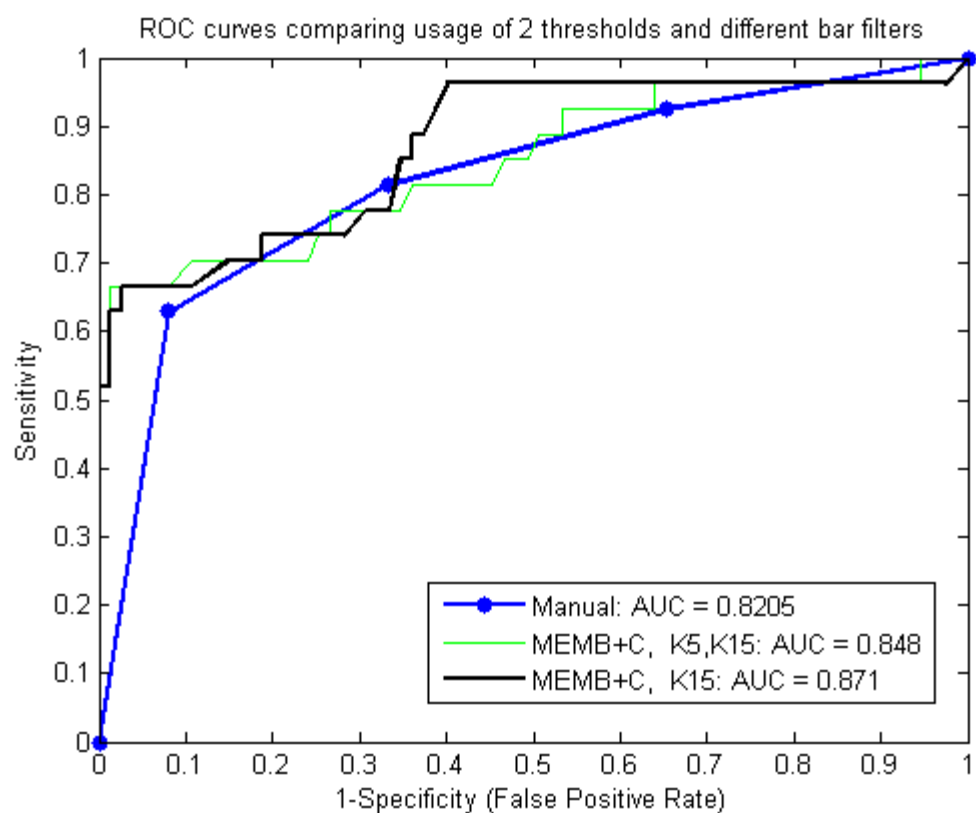

Supplement: Additional file 3 — ROC curves using Membrane Isolation Algorithm based on k = 15 and a combination (k = 15, k = 5). This is a comparison of ROC curves based on different Membrane Isolation Algorithm (MIA) variations. Since lighter cases showed enhanced membrane isolation detection when lower thresholds were used, a MIA using k = 5 threshold for lighter images and k = 15 for darker images was evaluated (green line). However, this did not improve AUC in comparison to one universal threshold (black line), and consequently, only one threshold (k = 15) was used in the results of this manuscript. It is interesting to note that the combination threshold used had very similar results to manual scoring (blue line). [file 1471-2342-8-11-S3.pdf]
